# Prevalence of addictive behaviors and mental health problems among Tunisian high school students

**DOI:** 10.1192/j.eurpsy.2026.10889

**Published:** 2026-07-31

**Authors:** S. Ben Fredj, R. Ghammam, N. Zammit, A. Skhiri, O. Ben Saad, F. Chouikha, A. Chouchen, J. Maatoug, I. Harrabi

**Affiliations:** 1 Department of Preventive Medicine and Epidemiology, “LR19SP03”, University Hospital Farhat Hached; 2 Faculty of Medicine of Sousse, University of Sousse; 3 Department of Occupational Medicine, “LR19SP03”, University Hospital Farhat Hached , Sousse, Tunisia; 4 Community and Preventive Medicine Department, Primary health Care Corporation, Doha, Qatar

## Abstract

**Introduction:**

Adolescence is a critical developmental stage often marked by the initiation of risk behaviors and the emergence of psychological difficulties. Tobacco, alcohol, and drug use remain major public health concerns worldwide. In contrast, behavioral addictions such as problematic Internet use and excessive video gaming are increasingly recognized as threats to adolescent health. In parallel, emotional and mental health challenges, including depression, anxiety, alexithymia, and low self-esteem, contribute to impaired well-being and long-term vulnerability.

**Objectives:**

To determine the prevalence of addictive behaviors and mental health among Tunisian high school students.

**Methods:**

We conducted a cross-sectional study in the urban area of the Sousse governorate in Tunisia during the 2018/2019 school year. We selected a representative sample of high school students enrolled in public educational institutions in Sousse. Data collection was performed through a self-administered structured questionnaire, which gathered information on sociodemographic characteristics, lifestyle behaviors, and mental health disorders (Depression, Anxiety, Self-esteem, Alexithymia, Problematic use of Video Games and Facebook) using validated Arabic version scales. Statistical analysis was carried out using the SPSS program (version 20).

**Results:**

We enrolled a total of 1342 high school students in our study, of whom, 63.2% were female with a mean age of 17.5±1.44 years. The prevalence of tobacco use was 16.5%. E-Cigarettes were the most used tobacco product among high school students, with a prevalence of 11.1%, and 27% of adolescents acknowledged having smoked at least once in their lifetime. The prevalence of drug use and alcohol use was 9% and 6.1% respectively. Boys were more likely to use drugs than girls (11.9% vs. 7.4%, p = 0.005). The prevalence of Facebook and Video game addiction was 28.3% and 13% respectively. Boys were more likely to be addicted to video games than girls (24.1% vs. 6.8%, p < 0.001). Girls were more likely to be addicted to Facebook (18.3% vs. 34.0%, p < 0.001). A notable proportion of adolescents (66.8%) manifested signs of alexithymia, 11.4% of the participants presented severe depression, and 39% of students reported experiencing low or very low levels of self-esteem. More than half of the participants (67%) demonstrated symptoms of anxiety disorders.

**Conclusions:**

These findings highlight the substantial burden of risk behaviors and mental health problems among adolescents, with notable gender differences across several domains. The high prevalence of tobacco experimentation, alcohol and drug use, digital addictions, emotional difficulties, and physical inactivity underscores the urgent for integrated prevention and intervention strategies targeting both behavioral and psychological well-being in this vulnerable population.

**Disclosure of Interest:**

None Declared

